# An Analysis of Drug Use-Related Curriculum Documents for Paramedic Students in British Columbia

**DOI:** 10.7759/cureus.48515

**Published:** 2023-11-08

**Authors:** Jennifer L Bolster, Alan M Batt

**Affiliations:** 1 Department of Paramedicine, Monash University, Melbourne, AUS; 2 Department of Clinical Governance and Professional Practice, British Columbia Emergency Health Services, Vancouver, CAN; 3 Faculty of Health Sciences, Queen's University, Kingston, CAN

**Keywords:** education, pre-hospital care, paramedicine, emergency medical services, substance use disorder, emergency health services, addiction

## Abstract

Background and aims: Paramedics attend an unprecedented number of drug poisoning events daily in British Columbia (BC), Canada, due to the ongoing public health crisis related to an increasingly toxic and unregulated street supply of illicit drugs. Paramedics have the potential to support alternative models of care to reduce harm, but their perspectives toward harm reduction initiatives are polarized. Understanding the drug-related substance use content in paramedic curriculum documents is important for deploying effective harm-mitigating programs. The aim of this study was to determine how illicit drug-related substance curriculum prepares paramedics for practice in British Columbia.

Methods: We performed a document analysis of curriculum documents in BC’s paramedic training institutions, the primary program textbook, and the 2011 National Occupational Competency Profile (NOCP) for Paramedics in Canada. We used O’Leary’s eight-step process to guide the planning and procedure of the analysis. We analyzed and coded documents both inductively and deductively and subsequently combined, refined, and used the codes to inform the development of themes via reflexive thematic analysis. The Checklist for Assessment and Reporting of Document Analysis (CARDA) tool was used to report our analysis.

Results: Of the 45 documents analyzed, 23 included codes relevant to the research questions. Paramedics are primarily taught to care for people who use drugs in an acute drug poisoning response only, with little consideration of holistic care and no meaningful mention of harm reduction. Some stigmatizing language was found within the content.

Conclusions: Many opportunities to introduce holistic models of care for people who use drugs along the entire continuum of care are unaddressed by paramedic curriculum documents in BC. Curriculum developers should include people who have lived and living experience of drug use in the co-design of educational programs involving their care. Further qualitative analyses are required to evaluate the relationship between paramedic education and provider-based stigma.

## Introduction

Paramedics in British Columbia (BC) manage an incidence of drug-related harm that is unmatched by any other paramedic services in Canada [1]. In 2021, BC paramedics attended 35,525 drug poisoning events, a 31% increase from the previous year, and an 189% increase from 2015, prior to the announcement of a public health emergency by the provincial government [2]. Since this declaration, more than 12,000 BC residents have died due to illicit drug toxicity, and currently, drug poisoning deaths account for more deaths than homicides, suicides, drownings, motor vehicle incidents, and fire-related deaths combined [3].

Although paramedics play an important role in the resuscitation phase in their response to a drug poisoning event, their role in the post-resuscitation phase, where they must support the patient’s next steps in navigating the healthcare system, means they are uniquely positioned to reduce drug-related harm [4]. People who experience an out-of-hospital drug poisoning event who are not conveyed to the emergency department (ED) are at a significantly higher risk of short- and long-term mortality [5-7], and in BC, the incidence of non-conveyance to the ED is climbing significantly. As such, it has never been more important for paramedics to be empowered to act as system navigators, offering alternative destination pathways and concurrent harm reduction initiatives and programs [8]. Despite this, in some areas that have introduced harm-mitigating programs, paramedics appear to express polarizing views on their utility, describing sentiments that initiatives are not feasible and do not decrease drug-related deaths [9].

Further, paramedic students demonstrate significantly lower levels of empathy for people who use drugs than any other patient population, which in general tends to decline further as their training progresses [10-12]. This is a cause for concern, and it remains unclear what influence entry to practice education has on paramedics’ empathy toward people who use drugs. Empathy can be taught and developed, yet this is not routinely included in paramedic curricula in Canada [13].

What is included in paramedic education in Canada is an emphasis on linear responsive models that emphasize a public safety model and prioritize patient stabilization and transportation in the out-of-hospital setting [14]. The curriculum in general inadequately represents complex, contemporary practice, which is a cause for concern, considering the evolving demands of paramedic work, including their involvement in public health crises they were not best prepared for [15,16]. A narrow focus on the medical aspects of drug use ignores the complex social and structural determinants of health that influence drug use patterns and outcomes [17]. These individuals often face a range of intersecting health and social challenges, such as poverty, mental illness, social isolation, and homelessness, that may exacerbate their drug use and increase their risk of drug-related harm [18].

Therefore, a more comprehensive, holistic approach that addresses the complex health and social needs of people who use drugs is necessary [4]. We aimed to investigate paramedic education and determine whether current paramedic education in BC adequately prepares paramedics to provide holistic care for people who use drugs. Identifying gaps in paramedic education is crucial to enable paramedics to play a more significant role in reducing drug-related harm in BC and align service delivered with health system expectations.

## Materials and methods

Rationale

We elected to conduct a document analysis following a conversation in which we explored paramedic attitudes toward harm reduction initiatives and low student empathy scores toward people who use drugs. Further, we noted in the literature that practicing paramedics appeared polarized toward the idea of integrating harm reduction initiatives into their practice [19,20]. To better understand where these perspectives are potentially formed, we sought to evaluate entry-to-practice paramedic education and the documents that shape and inform such education. The protocol for this study can be found at http://osf.io/sr9wj.

Document corpus

Documents were eligible for inclusion if they involved guiding curriculum or standards that inform didactic learning for primary and advanced care paramedics in British Columbia. Existing curriculum documents from the schools included lesson plans, course schedules, curriculum guides, simulation packages, lesson supplementary information, PowerPoint slide decks, and instructor notes. The target audience for all included documents were paramedic students.

Existing curriculum documents from the two major paramedic training institutes in British Columbia, the Justice Institute of British Columbia (JIBC) and Columbia Paramedic Academy, were included. Both the Primary Care Paramedic (PCP) and Advanced Care Paramedic (ACP) program curriculum documents were included for analysis; however, we were unable to obtain documents for one of the school’s ACP programs, as it is a relatively new program (see Limitations). To maintain focus on the research questions, the education institutions will remain anonymous in the results.

The curriculum documents from the schools were created by program lead instructors and curriculum designers. In addition, we analyzed the primary paramedic textbook for both schools: Nancy Caroline’s Emergency Care in the Streets (Eighth Edition) [21] and the national accreditation guidance document, the Paramedic Association of Canada’s 2011 National Occupational Competency Profile (NOCP) [22]. The NOCP outlines the competencies of paramedic practice within Canada, and although not intended to be a curriculum blueprint, it serves as a guiding framework for many educational institutions to inform curriculum design. The NOCP is currently undergoing a comprehensive revision and is set to be released in early 2024 (see Appendices for the table of documents).

Document collection and management

Curriculum documents from the paramedic institutes were requested and obtained via e-mail in August 2022. The 2011 NOCP document is publicly available at paramedic.ca/competencies/nocp and was downloaded onto the primary researcher’s computer via the Internet in August 2022. We acquired an online digital copy of Emergency Care in the Streets Eighth Edition from Jones and Bartlett Learning in July 2022. All documents were stored on the primary researcher’s password-protected computer and private folder.

Document quality

We found the majority of documents to be of reasonable quality. The NOCP and primary text documents specifically were complete and without redaction. The documents obtained from the two paramedic institutes were comprehensive in content; however, some documents lacked standardization. We found that all documents provided sufficient guidance in delivering lessons specifically, with evident flexibility for instructors to facilitate discussion among learners. No documents included any content that was redacted or obviously altered.

Preliminary data analysis

We conducted a first-pass review of the documents, as described by Bowen (2008) to ensure suitability for inclusion and to collect data to understand and analyze content [23]. The primary researcher read all documents and made annotations and notes within the research journal in NVivo (QSR International, Burlington, MA) to begin organizing the documents to prepare for coding.

Planning

We performed a document analysis utilizing O’Leary’s eight-step process for planning. Contingency planning was discussed early between us, and we decided that if one document was not available, the analyses would continue, but if two or more documents were not available, the scope of the work would be amended to appropriately address the research questions. We sought to determine the presence of certain words or concepts within texts or sets of texts [8] and the culture and time of which these are a part.

Procedure

We employed a summative approach to strengthen the analysis by providing insight into complex models of human thought and language [23]. We followed an eight-stage process when analyzing documents, which contained the steps outlined by O’Leary (2014), and reported our methods using the Checklist for Assessment and Reporting of Document Analysis (CARDA) [24,25]. This was done by counting and comparisons and habituality of keywords and content, followed by the interpretation of the basic context.

We developed 10 questions for interviewing the text as informed by O’Leary (2014). The development of the questions was reflexive in nature, and questions were adapted following a discussion between us after the initial analysis of the included documents. The questions were not adapted once coding commenced (see Appendices for the list of text interview questions applied as codes).

Analysis

The analysis of documents focused on both manifest and latent content, as well as the use of linguistics to evaluate the existence of any hidden or implicit messages. The first author (JB) systematically read the curriculum documents and coded all phrases relating to drug-related substance use both inductively and deductively. The coded data was reviewed by AB. We further coded questions against each document to gain a comprehensive understanding of the data (see Appendices).

We simultaneously made annotations to capture underlying tones or covert meanings within the texts. To ensure analyst immersion within the content, documentation of relationships, sentiments, common findings, and preliminary themes were recorded in a research journal, and we stored these as memos in NVivo version 12 (QSR International, Burlington, MA). We sorted text phrases of the documents that mentioned drug-related substance use (e.g., substance abuse, drug abuse, addiction, and substance misuse) into a language parent code to evaluate the potential existence of stigmatizing or outdated terminology. Phrases were considered holistic if they addressed the person who uses drugs with respect to the social determinants of health, not the drug use itself, and if the focus of care was placed outside of the response to the drug poisoning event alone. We iteratively interpreted the meaning of extracted phrases pertaining to drug-related substance use. Doubts concerning the inclusion of codes, text phrases, and their position within the thematic analysis were journaled and discussed between us. We undertook reflexive thematic analysis informed by Braun and Clarke (2018) by gaining familiarity with the data in the form of visual data representation, followed by the grouping and collapsing of codes in the remaining phases, with consultation between us during each phase of the analysis, ensuring trustworthiness standards were met throughout [26]. A table that represents the third phase of our thematic analysis where we generated initial themes based on the presence of codes can be found in the Appendices.

Measures to ensure trustworthiness

To ensure rigor and trustworthiness in the validity of our research, we assessed the potential risk of bias and evaluated the quality of the documents. We explored the document’s agenda and possible sources of bias and assessed the documents based on criteria such as relevance and reliability as described by O’Leary (2014). By following O’Leary’s process for conducting document analysis, while utilizing the CARDA reporting checklist, we ensured that our document analysis design was systematic and rigorous. We aimed to ensure credibility through prolonged engagement with the data. Raw data were stored and organized in archives. Efforts to maintain dependability included documented reflexive journal entries, annotations, and a well-described audit trail of code genesis, which included saved notes that highlight the rationale for code inclusion and exclusion. Confirmability was ensured by a clear audit trail of codes and detailed notes regarding the study characteristics, concepts, and themes, all of which were underpinned by a reflexive approach [27].

Reflexivity and positionality

The lead researcher (JB) is a Caucasian, female, postgraduate-educated paramedic, and subject matter expert on the paramedic response to the drug poisoning crisis in British Columbia. She is a leader of clinical governance and professional practice within her organization, with 10 years of experience. AB is a Caucasian, male, doctoral-educated paramedic researcher with over 25 years of experience in out-of-hospital care and methodological expertise including document analysis. We approached this study from a phenomenological lens, aiming at construing the meaning of the document on a surface level and any underlying meaning. We have experience as educators collectively with seven different institutions in Canada, including the two institutes studied. We do not personally have lived or living experience of drug use and addiction, but we have experience working with people who use drugs. Together, our approach to this analysis aimed to employ both meaningful interpretation and documented objectivity to inform the analysis of the documents.

## Results

A total of 45 documents were identified, procured, and analyzed, comprising 43 curriculum documents, the 2011 NOCP, and the textbook. For the NOCP, we analyzed one document (NOCP main document), and for the textbook, we analyzed a total of five chapters: Chapter 3 (Public Health), Chapter 13 (Principles of Pharmacology), Chapter 14 (Medication Administration), Chapter 27 (Toxicology), and Chapter 28 (Psychiatric Emergencies). Text phrases pertaining to drug-related substance use were discovered in 22 of these documents. Only one of 22 documents described harm reduction as a component of paramedic practice. No documents included people who have lived and living experience of drug use in either co-design or review of the included documents.

**Figure 1:**
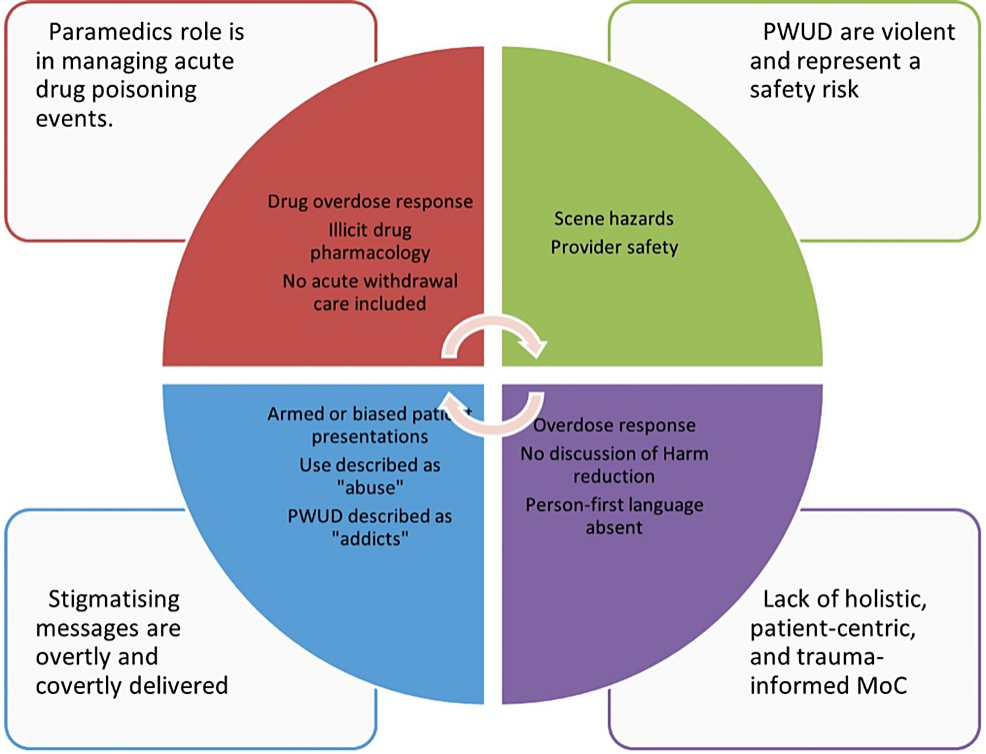
Thematic Analysis PWUD: people who use drugs, MoC: Models of Care

Using Braun and Clarke’s framework for reflexive thematic analysis [26], we developed four themes: The Paramedic Role: Acute Drug Poisoning Events Are the Only Time Paramedics Can Intervene, Patient Population: People Who Use Drugs Are Often Violent and Represent a Safety Risk to Paramedics, Words Matter: Stigmatizing Messages Are Overtly and Covertly Delivered to Paramedic Students, and Models of Care: Lack of Holistic, Person-Centered, and Trauma-Informed Practices (Table 1, Figure 1).

**Table 1:** Quotes Corresponding With Themes ABC: airway, breathing, circulation, EMS: emergency medical services

Theme	Quotes
The Paramedic Role: Acute Drug Poisoning Events Are the Only Time Paramedics Can Intervene	“The rapid recognition of opioid overdose followed by appropriate treatment,” “As a paramedic, you may be unable to explore all these areas during a short transport to the medical facility, particularly because much of your time will be devoted to ensuring the safety of your crew and managing the patient’s ABCs” (5, page 1476)
Patient Population: People Who Use Drugs Are Often Violent and Represent a Safety Risk to Paramedics	“Aggressive and dangerous behaviors are often caused by the use of illicit drugs” (5, page 1466), “Be aware that patients who have taken an overdose may be extremely dangerous” (5, page 1406), “Their behavior can quickly become violent, so always be mindful of your exit strategy when on scene. Do not hesitate to ask for law enforcement support if the scene seems likely to destabilize” (5, page 1414), “In addition to the threat from bystanders, the risk of a patient becoming aggressive is always present, particularly when cocaine or methamphetamines are involved” (5, page 507), “Such people are often paranoid, emotionally unstable, and almost always armed, making them a far more serious threat than an average patient with a non-drug-induced behavioral emergency” (5, page 507)
Words Matter: Stigmatizing Messages are Overtly and Covertly Delivered to Paramedic Students	“Understanding the complex nature of substance-related disorders is your first step in providing professional, competent, and compassionate care to all affected people, from the homeless drug addict to the substance-dependent businessperson” (5, page 1476), “Human beings have a long history of abusing drugs,” “You are almost certain to encounter patients who abuse medications” (5, page 639), “Discuss stigma and mental health associated with addiction,” “Watch Video: ‘Bringing Out the Dead,’ Discussion: Professional or not?” “A person with a drug addiction experiencing an acute psychotic break poses its own unique threats” (5, page 1457)
Models of Care: Lack of Holistic, Person-Centered, and Trauma-Informed Practices	“When it comes to drug use and overdoses, EMS has always been ‘reactive.’ Someone overdoses, we give Narcan, transport” (5, page 81), “Determining the most effective treatment for substance-related disorders requires an integrative approach of examining the social, biologic, cultural, cognitive, and psychological dimensions of the problem” (5, page 1476), “Discussion about Addiction vs. Dependence in context of opioids and alcohol,” “Apart from the physical effects of substance abuse, addiction carries a social stigma that can lead to feelings of isolation, paranoia, and depression” (5, page 1403), “I decided that, as the organization that sees these situations firsthand, we should be part of the conversation, and hopefully, have a hand in developing a meaningful solution to the problem that is plaguing our county” (5, page 81)

The Paramedic Role: Acute Drug Poisoning Events Are the Only Time Paramedics Can Intervene

**Figure 2:**
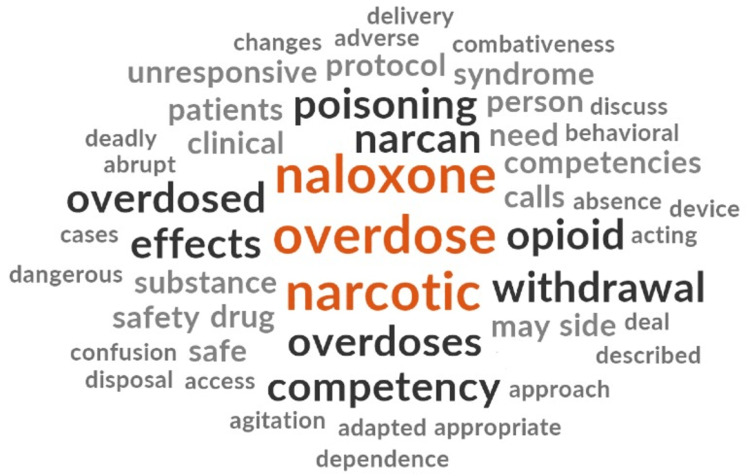
Word Cloud for Theme 1

There was a universal focus on resuscitation as the primary paramedic role in caring for people who use drugs. From a clinical perspective, no content addressed the treatment of a patient in acute illicit drug withdrawal or other aspects of care for people who use drugs such as wound care or offering harm reduction supplies or alternative care and destination pathways following the resuscitation phase. Phrases and words were extracted if they pertained to care provided during an acute drug poisoning event (Figure 2).

The 2011 NOCP included no competencies related to drug-related harm reduction. Within the “Toxicological Illness” section of the NOCP, addiction, drug poisoning prevention, harm reduction, and acute withdrawal were not described. The phrase “toxicological syndromes” was used throughout pertaining specifically to drug poisonings and did not discuss or reference substance use or addiction outside of this context.

Other texts focused solely on patient assessment and treatment of a person experiencing a drug poisoning event with little mention of the paramedic role outside of this response. The drug poisoning response curriculum highlighted a step-based approach that focused on airway management, quality ventilation, and the administration of naloxone to restore patient respiration. Curriculum documents introduced students to drug paraphernalia, including different drug presentations, and drug supplies. Statements that included patient education and identification of “those at risk of opioid abuse” suggested that paramedics do have a greater role to play; however, these statements were not expanded upon.

The emphasis of drug-related response discovered within the texts is placed on opioid reversal and management, with significantly less attention on the care of people who use non-opioid illicit substances such as methamphetamine, cocaine, and other drugs. Care for special populations that include approaches to youth who use drugs or elderly who use drugs was not discovered in any of the documents.

Patient Population: People Who Use Drugs Are Often Violent and Represent a Safety Risk to Paramedics

**Figure 3:**
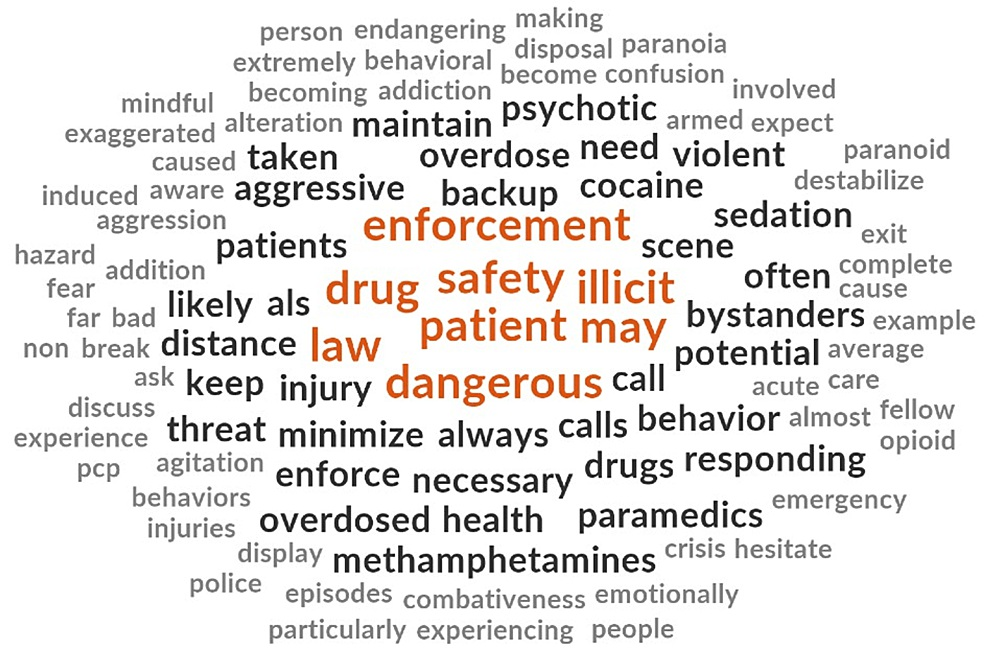
Word Cloud for Theme 2

Several phrases that spoke to the hazards and risks that paramedics may be exposed to while caring for people who use drugs were included (Figure 3). Although most scene hazards pertained to the risk of patient combativeness or violence, some documents included environmental hazards including exposure to sharps, such as used needles.

Many phrases made broad sweeping statements surrounding the relationship between illicit drug use and aggressive and dangerous behaviors (Table 1). Some statements imply stigmatizing assumptions broadly about people who use drugs such as their mental state and their inherent risk of threat.

Documents further described the relationship between patient violence and paramedic care and at times emphasized the risks that people who use drugs pose to paramedic safety by using inappropriate humor. One example was a PowerPoint slide that referenced a children’s book titled “Go to sleep” on the topic of people experiencing stimulant-related drug poisoning. The slide included expletives and implied that people experiencing stimulant poisoning will likely need to be sedated by paramedics.

Words Matter: Stigmatizing Messages Are Overtly and Covertly Delivered to Paramedic Students

**Figure 4:**
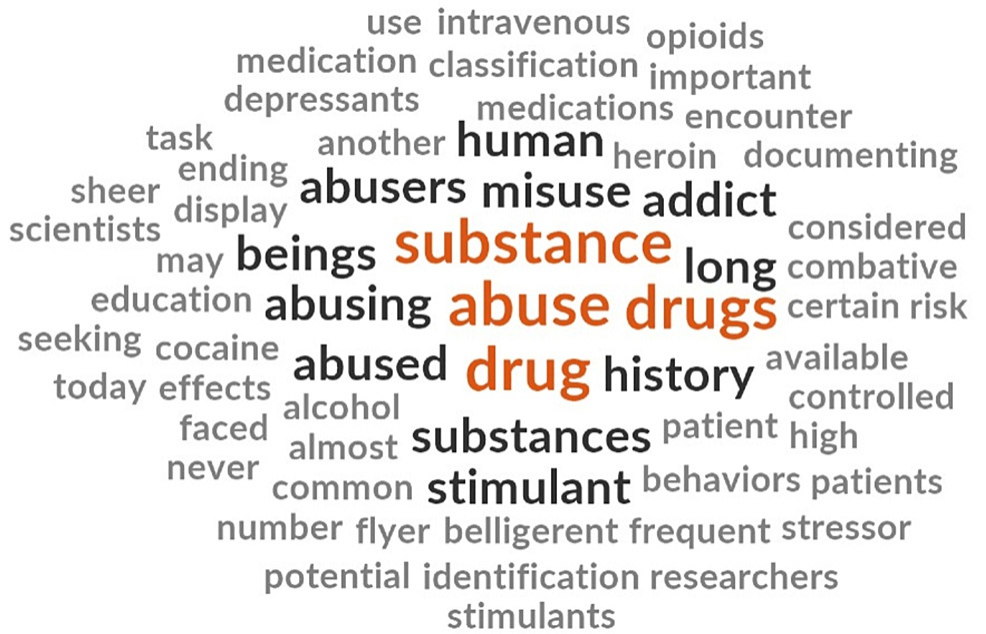
Word Cloud for Theme 3

Stigmatizing messages were discovered in the included documents. Specifically, messages included language no longer accepted as person-centered or trauma-informed, and covert assumptions or implicit statements were found (Figure 4).

Our analysis led to the discovery of inferences regarding people who use drugs who were often referred to as drug “abusers,” “misusers,” and “addicts.” Substance use was often referred to as substance “abuse.” “Drug-seeking” behavior is mentioned as common in people who use drugs, without much to qualify what this means in the out-of-hospital setting and its relevance to care provided by paramedics. In some instances, associations are made between socioeconomic status and substance use.

Despite this language, there are notes within the document that suggested facilitating classroom discussions regarding stigma and professionalism while caring for people who use drugs. This suggests that conversations are indeed taking place (or at least are intended to take place) surrounding the intersectionality between mental health, addiction, and drug-related stigma in the classroom. The conversations taking place and the conclusions those participating in them come to are unknown.

The use of person-first language was almost entirely absent from the included texts except for one statement. The statement also emphasized the risk this patient presentation poses to paramedics without any complementary statements explaining the causes of an “acute psychotic break” or how to care for this presentation.

Models of Care: Lack of Holistic, Person-Centered, and Trauma-Informed Practices

**Figure 5:**
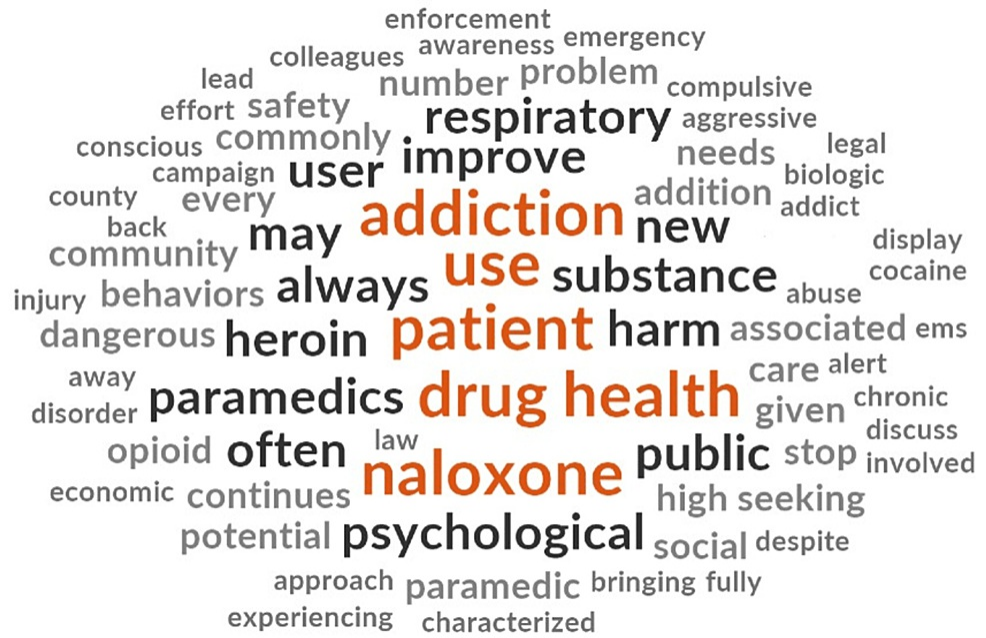
Word Cloud for Theme 4

Language within all included documents was found to be largely biomedical in nature. Deviations from biomedical terminology existed in only eight phrases, which were coded as representing holistic care options. There was no content within any of the analyzed documents that referenced or provided information on trauma-informed care or practices (Figure 5).

Despite the language within the texts being predominantly biomedical, some holistic messages were observed. Examples of paramedics leading the expansion of their role to a more holistic entity are included in the reporting of a public awareness campaign named “Stop Heroin,” where one paramedic described her role as being limited by its responsive model (Table 1).

The documents hint at holistic and integrative approaches to the care of people who use drugs. Instead of exploring these approaches, however, responsive and resuscitative models of care are reemphasized, and at times, further care is downplayed as being outside of the paramedic’s scope.

Although there were statements within the documents that addressed titrating naloxone doses to avoid precipitation of acute withdrawal, the end goals appeared to be anchored in preventing the risk of violence and less about avoiding uncomfortable symptomatology.

## Discussion

Paramedic-led harm reduction approaches face barriers to their implementation due to stigma and low empathy scores among paramedic trainees for people who use drugs [11,13,21,22]. We sought to determine how illicit drug-related substance curriculum prepares paramedics for practice in British Columbia. We analyzed the curriculum from two paramedic education institutions, a core textbook, and a national competency document. Our analysis highlighted that the paramedic role is described as limited to drug poisoning response and management, people who use drugs are often portrayed as violent and representing a safety risk to paramedics, stigmatizing messages are overtly and covertly delivered to paramedic students, and there is a significant lack of holistic, person-centric, and trauma-informed practices introduced to paramedic students within the intended curriculum.

While opioid toxicity resuscitation and drug poisoning response remain essential, paramedics are increasingly responding to events where co-intoxicants complicate care, leading to multiple high doses of naloxone administration, and unresolved coma, despite complete opioid toxidrome reversal [28-30]. Notwithstanding drug contamination, out-of-hospital treatment of a drug poisoning event has proven insufficient in reducing drug-related harm and mortality [31,32]. This is evidenced by soaring death tolls as reported in the British Columbia Coroner Service (BCCS) Death Review Panel, which highlighted that illicit drug toxicity is the leading cause of unnatural death in the province [3]. Equally alarming is the climbing frequency of non-conveyance to the ED by paramedics following a drug poisoning event and the correlating increases in the risk of short- and long-term mortality [6,8]. Lending to concerns around non-conveyance is the decreasing prevalence of 911 activation by the community of people who use drugs [33].

As the degree of drug toxicity and drug-related mortality increases, while the incidence of 911 calling and ED conveyance decreases, the opportunity for paramedics to enact holistic models of care that include harm reduction programs is narrowing. A shift is required to recalibrate the focus of the paramedic role in caring for people who use drugs, one that is currently not reflected in (and may indeed be hindered by) paramedic student education as explored in this analysis. The description of these responsive models of care is further limited by the focus placed on resuscitation. Under-explored areas of response include care of people who use non-opioid illicit substances, targeted approaches to care of youth, elderly, and indigenous patients, and care of patients in acute withdrawal.

Missing entirely from the findings of our analysis is the opportunity to expand on the downstream negative effects of leaving a patient who does not wish to be conveyed to the ED in acute withdrawal. The pathophysiological milieu that manifests as a person is placed into acute withdrawal and how this impacts a paramedic’s perception of their presentation is worthy of consideration in the curriculum. Similar attention should be placed on why these symptoms arise and how, for example, neurochemical changes that take place may be perceived by the paramedic as violent and aggressive predispositions. It was also noted that opportunities to introduce students to alternative models of care such as take-home naloxone kit delivery, referral pathways, and alternative destination pathways were missing from the documents. Alternative and holistic models of care are increasingly being recognized as crucial, given the frequency at which paramedics interact with people who use drugs [4]. Further, despite the importance of trauma-informed approaches required to provide holistic care for people who use drugs specifically, no content introduced this concept.

The increasing incidence of violence against paramedics and occupational risks has been described as a serious public health problem [34-37]. While paramedic safety should take utmost priority, curriculum developers must be mindful of their choice of language, being careful not to use language that generalizes or makes assumptions about an entire patient demographic [38,39]. The use of absolute and definitive language around patient violence has the potential to create negative associations for paramedic students. Statements such as “these patients are almost always armed” (5, page 1466) or “aggression is almost always caused by drugs” (5, page 1466) have the potential to cause preconceived ideas that may strain the relationship between the paramedic and people who use drugs prior to any actual patient encounter. Because of these preconceived notions regarding violence, paramedics may begin their interaction with people who use drugs preparing for such an encounter, potentially leading to a demeanor that could be inadvertently perceived by people who use drugs as negatively authoritative or non-empathetic [40]. Of course, it is not words alone that influence how communication is perceived; negative tone, closed-off body language, or general approach that may come off as disapproving or judgmental may all contribute to a negative interaction, regardless of the words used themselves [41]. Additional education related to communication and the importance of language with special respect to those experiencing further marginalization (e.g., indigenous peoples, LBTQIA2S+, and unhoused) who may have had previous negative interactions with the public safety or healthcare system may be beneficial. Without such additional education, paramedics may be unaware of how their language is being perceived. Not only are students being taught a medical language by which to communicate, but they are also being taught occupational language norms by which they will eagerly adapt to their own vocabulary.

The utilization of person-first and inclusive language creates an environment where people feel like they can seek assistance when required [42]; however, we found that the use of person-first language within this analysis was almost non-existent. Inversely, when people are spoken to or about in ways that may be perceived as dehumanizing, they are less likely to reach out for help and are more likely to use drugs alone, placing them at higher risk [43]. It is important to consider that not all stigmatizing language is intentional or overt and that the intent of the message is not always the impact, especially in patients who may have long-standing experiences of intergenerational trauma and discrimination [44]. The use of person-centric and trauma-informed language will be essential in the shift toward holistic models of patient care delivery and should be reflected in curriculum documents [45].

By introducing such concepts into paramedic education, we can begin to offer opportunities for paramedics to positively influence the journey of people who use drugs through the healthcare system. Initiatives such as alternative care pathways, take-home naloxone programs, and treatment of acute withdrawal have the potential to not only reduce patient harm but also reduce the harm bestowed upon paramedics performing a role that is increasingly perceived as restrictive, limiting, and ineffective. The effects these models of care have on resilience, compassion, and empathy have been described in qualitative analysis across the province of BC. Locally, this was demonstrated by Williams-Yuen et al. (2020), who evaluated the ways BC paramedics experience the overdose crisis [32]. Paramedics described an emotional burden associated with the ongoing crisis, correlated directly with one’s capacity to help. Because paramedics begin expressing diminished levels of empathy for people who use drugs long before they begin clinical practice, it is timely to ensure the curriculum integrates education on empathy, emotional burden, and moral distress, and we suggest holistic models of care that involve harm reduction may combat many of these distressing associations.

Involving people who have lived and living experiences of drug use in the co-design of programs that involve their care is a meaningful way to address gaps in curriculum design [46-48]. Patients, as the end users of paramedic services, play an important role as stakeholders in directing their goals of care more broadly. Engaging the patient voice in health professions education can enrich the educational experience while promoting justice and empowering a compassionate approach among healthcare providers [49]. Efforts to engage patients should be considered early to avoid any unintended tokenistic views or perceptions. Engagement with marginalized and underserved populations should be driven by principles of ethical engagement and, in general, should be compensated opportunities [50].

Limitations

The ability to analyze what paramedics in BC are taught about drug-related substance use is limited by the nature of our analysis of didactic learning documents. In academia lives the intended curriculum, the enacted curriculum, and the experienced curriculum, which vary in content [51]. This paradigm of what lives within curriculum documents versus what is taught is a complex commonality of health education and culture, the scope of which lies outside the purpose of this study. Without observing the context, tone, language, instructor biases, supplementary information, deviations from curriculum guidance, or sidebar conversations delivered to paramedic students by lecturers, it is impossible to conceptualize the full scope of what paramedics learn about drug-related substance use. It is also important to recognize that paramedic education extends far past what is learned in the classroom and that what is learned via clinical placement and practicum requires urgent attention. We further recognize our positionality as people who do not have lived and living experience of drug use in analyzing the content being a limitation in itself. Although time constraints prohibited us from including a person who has lived experience with drug use in this study, we recommend including this important perspective to strengthen future analysis. In addition, we were unable to collect drug-related curriculum documents from one of the schools’ ACP programs due to the curriculum being under development and not finalized or prepared for release.

## Conclusions

Our analysis of paramedic curriculum documents in BC has highlighted the presence of stigmatizing language and negative preconceptions toward people who use drugs, which may be hindering the implementation of paramedic-led harm reduction approaches. The paramedic role is currently described as limited to drug poisoning response, which is insufficient to reduce drug-related harm and mortality. The increasing degree of drug toxicity and mortality and decreasing incidence of 911 calling and ED conveyance necessitates a shift toward holistic models of care. The language used in paramedic education needs to be mindful and inclusive to avoid preconceived notions and negative associations and to facilitate positive interactions with people who use drugs.

The importance of contemporary drug-related substance use education for paramedic students is becoming apparent in the context of the ongoing public health crisis related to an increasingly toxic supply of drugs in BC. Future drug-related substance use curriculum development should aim to reflect the evolving role of the paramedic in the drug poisoning crisis. Academic program design leaders should consider the ethical engagement of people who have lived and living experience of drug use as stakeholders in the co-design and potentially co-delivery of a curriculum that involves their care. Harm reduction and the many opportunities that paramedics have to positively influence a patient’s journey through the healthcare system should receive equal attention in the curriculum to that afforded to the emergency drug poisoning response.

## Appendices

Table 2 represents the third phase of our thematic analysis where we generated initial themes based on code frequency.

**Table 2:** Initial Generation of Themes

Name of Parent and Child Codes	Files	References
Defining Substance Use and Addiction	0	0
Addiction	4	11
Addictive	1	3
Dependence	1	7
Habituation	4	4
Tolerance	1	1
Drug User	1	1
Substance Use	3	4
Substance-Related Disorder	1	4
Alcohol or Cigarette Use	1	2
Social Determinants of Health	1	2
Stigma	3	3
Withdrawal	4	5
Illicit Drug Pharmacology	0	0
Drug Classification	0	0
Benzodiazepine	1	3
Carfentanil	1	2
Cocaine	7	25
Crack	1	3
Ecstasy	1	1
Fentanyl	5	6
Heroin	1	1
Methamphetamine	5	21
Narcotic	6	47
Opiate	4	4
Opioid	11	33
Stimulants	8	16
Illicit substances	3	6
Embedded Stigma	0	0
Stigmatizing	5	16
Addict	1	2
Drug Abuse	8	23
Drug Abuser	1	2
Drug-Seeking	1	1
Frequent Flyer	1	1
Misuse	2	3
Substance Abuse	6	43
Triggering	2	2
Interview Questions	0	0
Audience	1	1
Interpretation of Text	2	4
Intended Audience	5	25
Non-intended Audience	4	12
Purpose of Text	3	3
Text Author	2	2
When Text Produced	1	3
How Text Reflects Time Written	0	0
Why Text Included	2	5
Language	1	3
Biomedical	3	7
Covert Assumptions and Overt Messages	4	21
Holistic	4	7
Patient-Centered	0	0
Trauma-Informed	0	0
Person-First	1	1
Tone	0	0
Negative	3	12
Neutral	1	1
Positive	1	1
Overdose Response	0	0
Competencies	4	9
Drug Poisoning	1	4
Narcan or Naloxone	12	55
Opioid Antagonist	1	1
Opioid Overdose	2	9
Overdose	15	113
The Paramedic Role in the Toxic Drug Crisis	0	0
Competencies	4	9
Opioid Epidemic	1	4
Context of Practice	1	2
Harm Reduction	1	2
Threat to Paramedics	4	21
Police	2	2

The codes applied to each document are expressed in Table 3. A full list of all 45 documents included in the analysis can be accessed through the primary researcher.

**Table 3:** Documents Included in the Analysis PCP: Primary Care Paramedic, NOCP: National Occupational Competency Profile, ACP: Advanced Care Paramedic

Document Types	Document Name	Codes	Number of Provided Documents
PDF Textbook	Nancy Caroline’s Emergency Care in the Streets (Eighth Edition)	271	1
PowerPoint Slide Deck, Learning Objectives, Assignment Documents (Worksheets), Course Outline, Teaching Target Points, Assigned Reading Resources	PCP Program (de-identified)	233	19
Lesson Plans, Simulations, Course Outlines, Instructor Guides, Practice Guidelines, Workshop Activities, Assignments, Study Guide	PCP Program (de-identified)	212	18
PDF Competency Profile	2011 NOCP	85	1
PowerPoint Slide Deck, Lesson Plans, Study Guide, Additional Reading Resources	ACP Program (de-identified)	82	5
PDF Assigned Reading Resource	Acute Poisoning Emergencies	40	1
